# Supplementary material for: Identification of photoperiod-induced specific miRNAs in the adrenal glands of Sunite sheep (Ovis aries)
Source: Front Vet Sci. 2022 Jul 22;9:888207. doi: 10.3389/fvets.2022.888207 (PMC9354845; doi:10.3389/fvets.2022.888207)
Supplement: Supplementary file 1 [file Data_Sheet_1.ZIP › Supplementary file/miRDeep2 file.zip!/miRDeep2 file/Know_Structure/SPLP42A1/oar-mir-379.pdf]

miRBase precursor : oar-mir-379
Total read count : 38984
oar-miR-379-5p read count : 36738
oar-miR-379-3p read count : 2235
remaining reads : 11

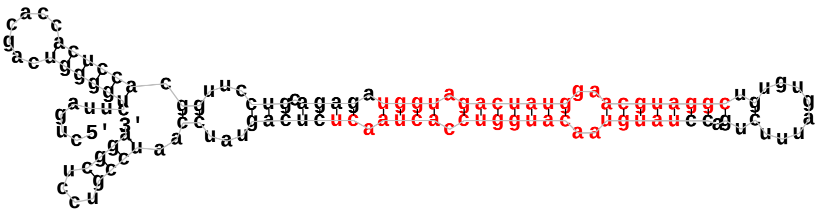

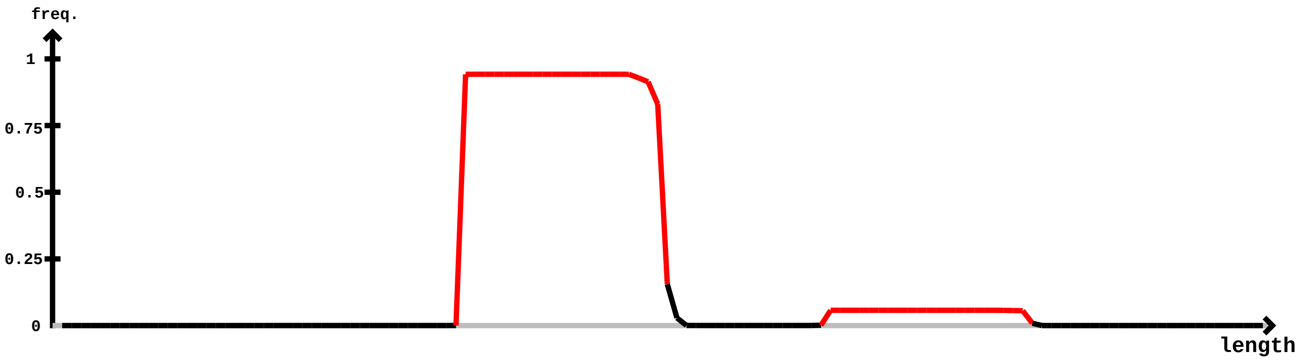


```
                                                                                               oar-miR-379-3p
                                            oar-miR-379-5p
5'- cugauucuggggucagcaccacuccacgguuccugcagagaugguagacuauggaacguaggcugugugauuucugaccuauguaacaugguccacuaacucucaguauccaauccguccucgga   -3'    exp
    .......((((((.......)))))).((...(((.((((.((((.(((((((..((((((((.(........).).)))))))..))))))).)))).)))))))...))..((((....))))    reads    mm
    .....................cuccacgguuccugcagaga....................................................................................    2        0
    ........................................augguagacuauggaacguag................................................................    1        0
    ........................................augguagacuauggaacguagg...............................................................    3        0
    .........................................ugguagacuauggaacg...................................................................    8        0
    .........................................ugguagacuauggaacgu..................................................................    526      0
    .........................................ugguagacuauggaacgua.................................................................    569      0
    .........................................ugguagacuauggaacguag................................................................    3258     0
    .........................................ugguagacuauggaacguagg...............................................................    26319    0
    .........................................ugguagacuauggaacguaggc..............................................................    4927     0
    .........................................ugguagacuauggaacguaggcu.............................................................    1106     0
    .........................................ugguagacuauggaacguaggcug............................................................    5        0
    .........................................ugguagacuauggaacguaggcugu...........................................................    1        0
    .........................................ugguagacuauggaacguaggcugugug........................................................    2        0
    ..........................................gguagacuauggaacguagg...............................................................    11       0
    ..........................................gguagacuauggaacguaggc..............................................................    1        0
    ..........................................gguagacuauggaacguaggcu.............................................................    2        0
    ...........................................guagacuauggaacguagg...............................................................    1        0
    ..............................................................cugugugauuucugacc..............................................    6        0
    ..............................................................cugugugauuucugacU..............................................    1        1
    ..............................................................................cuauguaacaugguccacu............................    4        0
    ..............................................................................cuauguaacaugguccacua...........................    2        0
    ..............................................................................cuauguaacaugguccacuaa..........................    1        0
    ..............................................................................cuauguaacaugguccacuaac.........................    33       0
    ..............................................................................cuauguaacaugguccacuaacu........................    5        0
    ...............................................................................uauguaacaugguccacu............................    8        0
    ...............................................................................uauguaacaugguccacC............................    2        1
    ...............................................................................uauguaacaugguccacua...........................    21       0
    ...............................................................................uauguaacaugguccacuaa..........................    24       0
    ...............................................................................uauguaacaugguccacuaG..........................    5        1
    ...............................................................................uauguaacaugguccacuaac.........................    1788     0
    ...............................................................................uauguaacaugguccacuaacu........................    338      0
    ...............................................................................uauguaacaugguccacuaacuc.......................    2        0
    ................................................................................auguaacaugguccacuaac.........................    2        0
```
